# Characterization of antibiotic resistance genes and virulence factors in organic managed tea plantation soils in southwestern China by metagenomics

**DOI:** 10.3389/fmicb.2025.1580450

**Published:** 2025-05-01

**Authors:** Taobing Yu, Lang Cheng, Qing Zhang, Jida Yang, Huadong Zang, Zhaohai Zeng, Yadong Yang

**Affiliations:** ^1^State Key Laboratory of Maize Bio-breeding, China Agricultural University, Beijing, China; ^2^College of Agronomy and Biotechnology, China Agricultural University, Beijing, China; ^3^Agricultural Environment and Resources Institute, Yunnan Academy of Agricultural Sciences, Kunming, China

**Keywords:** organic management, antibiotic resistance genes, virulence factors, assembly process, soil microorganisms

## Abstract

Sustainable organic management practices have gained significant attentions for its potential health and environmental benefits. However, the spread of antibiotic resistance genes (ARGs) and virulence factors (VFs) in soils, plants, and agricultural products has severely limited the development of organic managements on agriculture. At present, the distribution and assembly of ARGs and VFs in organic managed tea plantation systems remains largely unknown. Here, we used metagenomic analysis to explore soil microbial taxa, ARGs and VFs in 20 years of conventional managed (CM) and organic managed (OM) tea plantation soils. Results showed that total abundance of ARGs in OM was 16.9% (*p* < 0.001) higher than that in CM, and the increased ARGs were *rpoB2*, *evgS*, *MuxB*, *TaeA*, and *efrA*. As for VFs, OM significantly increased the abundance of adherence, stress protein and actin-based motility compared to CM. Moreover, OM increased the relative abundance of soil microbial taxa harboring ARGs and VFs, which were *Streptomyces*, *Pseudomonas*, and *Terrabacter*, compared to CM. Network analysis suggested that OM increased the positive interactions of microbial taxa-ARGs, microbial taxa-VFs and ARGs-VFs compared to CM. Impact of stochastic process on the assembly of soil microbial taxa, ARGs and VFs in OM was stronger than that in CM. Overall, these findings provide a basis for integrating ARGs, VFs and pathogen hosts to assess the ecological and health risks in long-term organic managed soils, and increased efforts need to be done in reducing ARGs, VFs and bacterial pathogens in fertilizers for organic managements on agriculture.

## Graphical abstract

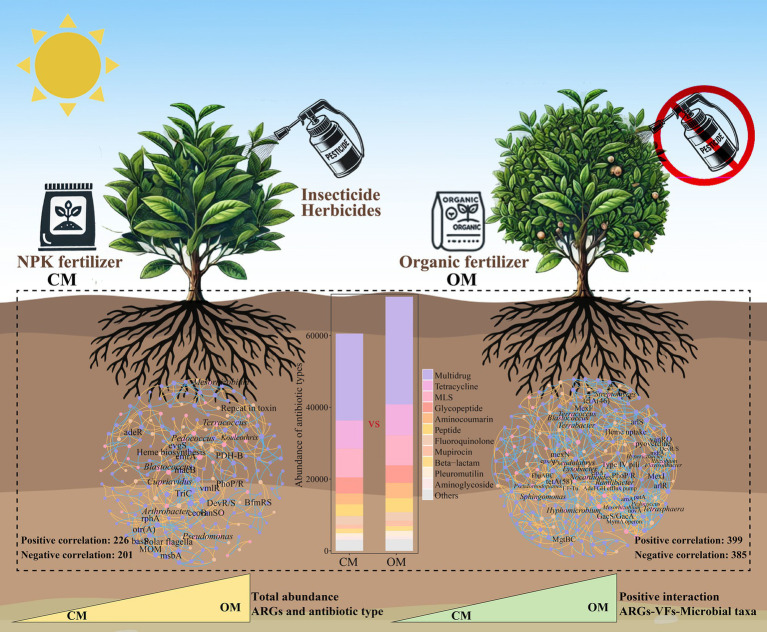

## 1 Introduction

In last decades, the widespread use and misuse of antibiotics in medical has led to the prevalence of antibiotic resistance genes (ARGs) in microbial communities (Kuppusamy et al., 2018; Wang J. H. et al., 2022). With the increase of ARGs and antibiotic resistant bacteria (ARBs) in the environment, antibiotic resistance poses a serious threat to ecological security and public health (Tiedje et al., 2019; Zhang et al., 2022). Soils are probably the most important hosts of ARGs, and a large number and diversity of ARGs have been found in soils around the world (Xiao et al., 2016; Braga et al., 2017). The ARGs in farmland soils can enter to plant stems, leaves and agricultural products, and further spread to humans along the food chain, posing a major threat to human health (Zhang et al., 2019; Song et al., 2023). Besides, the virulence factors (VFs) enable pathogenic bacteria to colonize a host, establish infection and confer virulence, thereby enabling its bacterial hosts to invade humans or animals and cause disease (Wu H. J. et al., 2008; Liang et al., 2020). When the ARGs coexist with VFs in the genome, the risk of the genome to humans or animals will increase (Liang et al., 2020). Many studies have reported that anthropogenic activities (such as irrigation, landfilling of waste, fertilization and cropping practices, especially organic fertilization) significantly enriched the abundance of ARGs in soils (Wang et al., 2014; Wu et al., 2017; Wang et al., 2018). Hence, it is urgent to assess to distribution and interaction of ARGs and VFs in organic managed farmland systems.

Tea (*Camellia sinensis* L.) plantations are widely distributed in tropical and subtropical acidic soils (Wang et al., 2016). Normally, high nitrogen fertilization rate was applied to obtain high yield in tea plantations (Yang et al., 2023), but its misuse may trigger negative environmental impacts (Wang L. L. et al., 2020). Organic management, such as using livestock manure, can reduce chemical fertilizer application, maintain soil fertility and improve soil biodiversity (Guo et al., 2017; Ekman et al., 2020). However, inputs of livestock-derived organic fertilizers may introduce ARGs and ARBs into farmland soils (Han et al., 2018; Sanz et al., 2022). Therefore, antibiotic resistance may spread from organically amended soils to crops, products and ultimately to consumers (Yang et al., 2018). Recent findings shown that the coexistence and convergence of ARGs and VFs in pathogenic bacteria significantly increased the risk of microbial contaminants in the environment (Yang et al., 2018; Liang et al., 2020; Li et al., 2023). Therefore, it is necessary to understand the characteristics of ARGs and VFs in long-term organic managed soils.

Studies have demonstrated that soil properties and microbial diversity are the main drivers that influence ARGs and VFs distribution (Nõlvak et al., 2016; Wei et al., 2022; Wu et al., 2023a; Wang L. et al., 2024). It is found that abundance and diversity of ARGs in soil are related to soil type and nutrient content in organic farming systems (Wang L. et al., 2022). Besides, soil physicochemical variables, such as soil organic carbon (SOC), total nitrogen (TN), soil TN:TP ratio and microbiomass-P, are strongly associated with the distribution and prevalence of ARGs or VFs in soils (Guo et al., 2020; Wang L. et al., 2020; Kang et al., 2023). Furthermore, soil microorganisms carrying ARGs can influence plant-associated microbiota through direct contact between the plant rhizosphere and bulk soil environment, and ultimately accelerate the evolution of ARGs in plant compartments (Chen et al., 2018). Environmental heterogeneity has been shown to determine the diversity and distribution of bacterial communities, and soil property variables induced by fertilization may indirectly influence ARGs distribution by shaping soil bacterial communities (Li H. et al., 2022; Wu et al., 2023b). Therefore, it is essential to elucidate the complexity and correlation of microbial communities and soil properties impacts on ARGs and VFs distribution in long-term organic managed soils.

Here, we used macrogenomic sequencing to analyze soil microbial communities, antibiotic resistance genes (ARGs) and Virulence factors (VFs) in conventional and organic managed tea plantation soils after 20 years. The aims of this study were (I) to investigate the effects of conventional and organic managements on soil microbial communities, ARGs and VFs; (II) to explore the biotic and abiotic factors that affect the composition and distribution of ARGs and VFs.

## 2 Materials and methods

### 2.1 Study site and soil sampling

Soil samples of the 0–20 cm layer were collected in July 2023 from a 20-year managed tea plantation (22.48°N, 100.58°E) in Pu’er City, Yunnan Province, China. The study site has a typical subtropical monsoon climate, with an annual mean precipitation of 1,311 mm and annual mean temperature of 21.5°C. Two treatments: conventional managed (CM, only NPK fertilizer) or organic managed (OM, livestock organic fertilizer) tea plantation soils with 20 years were selected in this study. The tea variety in the experimental area is Yunkang 10. The long-term experimental field was managed according to local practices, which usually received NPK fertilizer or sheep manure compost for the past two decades. The organic management fertilization method includes basal fertilizer (November to December every year) and topdressing (May of the following year). The basal fertilizer was 12,000 kg ha^−1^ and the topdressing was 3,000 kg ha^−1^. The organic matter content, total nutrient content and pH of the organic fertilizer were 60, 5% and 7.5, respectively. In conventional management, the compound fertilizer (1,050 kg ha^−1^; N-P-K: 22-5-5) was applied in June each year, and Glyphosate and Diafenthiuron were used for weeding and pest extermination, respectively. In order to ensure the representativeness of the soil samples, we established 6 plots (20 m × 5 m) for each fertilization treatment to collect soil. Each plot used a five-point sampling method to collect soil, and five individual samples were mixed to obtain a duplicate sample. In total, 12 soil samples (2 treatments × 6 replicates) were obtained, then the soil samples were stored at 4°C and −80°C, respectively.

### 2.2 Analysis of soil properties and enzyme activities

Soil pH was determined using a pH meter (1:2.5, w/v). Soil organic carbon (SOC) and total nitrogen (TN) were determined by the K_2_Cr_2_O_7_ oxidation–reduction titration and Kjeldahl digestion methods (Bao, 2000), respectively. Soil ammonium nitrogen (NH_4_^+^-N) and nitrate nitrogen (NO_3_^−^-N) were determined using a microplate spectrophotometer (Thermo1510, Multiskan Go; Thermo Scientific Inc., Waltham, MA, United States). Activity of β-1, 4-glucosidase (BG), β-cellobiohydrolase (CE), β-xylosidase (BX), β-1,4-N-acetylglucosaminidase (NAG) and L-leucine aminopeptidase (LAP) were determined by a microplate spectrophotometer (Ex. 360 nm; Em. 450 nm; Thermo Scientific Inc., Waltham, MA, United States) using 4-methylumbelliferone (MUF) and 7-amino-4-methylcoumarin (AMC) coupled substrates (Marx et al., 2001).

### 2.3 DNA extraction, metagenomics sequencing and data analysis

Total microbial genomic DNA was extracted from 0.5 g soil using the E.Z.N.A.^®^ soil DNA Kit (Omega Bio-tek, Norcross, GA, United States), and the quality of extracted DNA was measured using NanoDrop® ND-2000 spectrophotometer (Thermo Scientific Inc., Waltham, MA, United States). The shotgunmetagenomic sequencing were performed using Novaseq6000 (Shanghai Majorbio Bio-pharm Technology Co., Ltd., Shanghai, China).

Raw sequences were trimmed and filtered using fastp version 0.20.0 software. Reads with average quality score lower than 20, containing more than three “N,” with length shorter than 50 bp and those reads matching the Illumina background sequences (artifact, spike-ins or phiX) were all removed. CD-HIT version 4.6.1 software was used for clustering, and the longest gene was selected as the representative sequence to construct a non-redundant gene set. Use BLASTP version 2.3.0 software to compare the non-redundant gene set with the NR database version 20,200,604, and obtain the species annotation results through the taxonomic information database corresponding to the NR database (Altschul et al., 1997).

### 2.4 ARGs and VFs analysis

We use the Comprehensive Antibiotic Resistance Database (CARD version 3.0.9) with Antibiotic Resistance Ontology (ARO) as its core for annotation of antibiotic resistance genes (ARGs) (Yang et al., 2022). The non-redundant gene sets were compared to the CARD database using BLASTP version 2.3.0 software, and the annotation of E to 1e^−5^ was selected. The setting parameters for ARGs annotation were ≥90% of sequence identity and ≥25 amino acids of alignment length. The ARGs obtained were classified by type (antibiotics to which the genes are resistant) and subtype (antibiotic resistance genes). To identify virulence factors (VFs) sequences in our data, open reading frames (ORFs) were compared against the virulence factor database (VFDB version 2020.07.03) using blastx with the *E*-value to 1e^−5^. The ORF with identity ≥90% and coverage ≥ 90% was annotated as a VFs (Liu B. et al., 2022; Liu W. B. et al., 2022). In addition, we annotated the species of ARGs or VFs to identify host bacteria.

### 2.5 Statistical analysis

Unpaired t tests were performed for significance analysis of two groups, and *p* values were adjusted by the false discovery rate test. Heatmap, boxplot and stacked chart were created using the OmicStudio.^1^ Principal coordinate analysis (PCoA) and redundancy analysis (RDA) were performed using the “vegan” package in R version 4.2.2 (Oksanen et al., 2013). Procrustes analysis was performed to examine the correlations between soil microbial communities, ARGs and VFs, and the sum of squares (M^2^) and *p*-value were used to determine the consistency of two datasets. The neutral community model (NCM) was performed using the “hmisc” and “minpack.lm” packages in R version 4.2.2 to evaluate the impact of stochastic dispersal on the assembly of soil microbial communities, ARGs and VFs (Ning et al., 2019). We selected soil microbial taxa, ARGs and VFs with relative abundance greater than 0.1% to construct the co-occurrence network. The correlations were computed using the “Hmisc” package in R version 4.2.2, with a strict absolute value threshold set to 0.9. To increase the credibility of the network analysis, only correlations with adjusted *p* values less than 0.01 were retained. Network visualization was performed using the Gephi version 0.9.2 software (Bastian et al., 2009; Yu et al., 2023).

## 3 Results

### 3.1 Soil properties and enzyme activities

Soil properties and enzyme activities varied greatly in OM and CM soils (Supplementary Table S1). The soil pH, TN content and SOC content in OM was 29.9, 9.3 and 6.6% (*p* < 0.05) higher than that in CM. The soil activities of NAG and LAP in OM was 26.8 and 184.1% (*p* < 0.05) higher than that in CM. However, no difference was detected in content of NO_3_^−^-N and NH_4_^+^-N, and activity of BG and CE between OM and CM (Supplementary Table S1).

### 3.2 Microbial diversity and community composition

A total of 6.1–7.6 Gb high quality clean reads was obtained after quality control for each sample (Supplementary Table S2). The filtered sequences were assembled *de novo* and 233,226 to 550,838 sequences were obtained, with a 481–598 bp for N50 and 332–345 bp for N90, for each sample. Each sample has 264,897–678,746 ORFs with a mean 373–419 bp per sample (Supplementary Table S3). Furthermore, bacteria and archaea dominated in total sequences with proportion of 65.3 and 19.5%, respectively (Supplementary Table S4).

**Figure 1:**
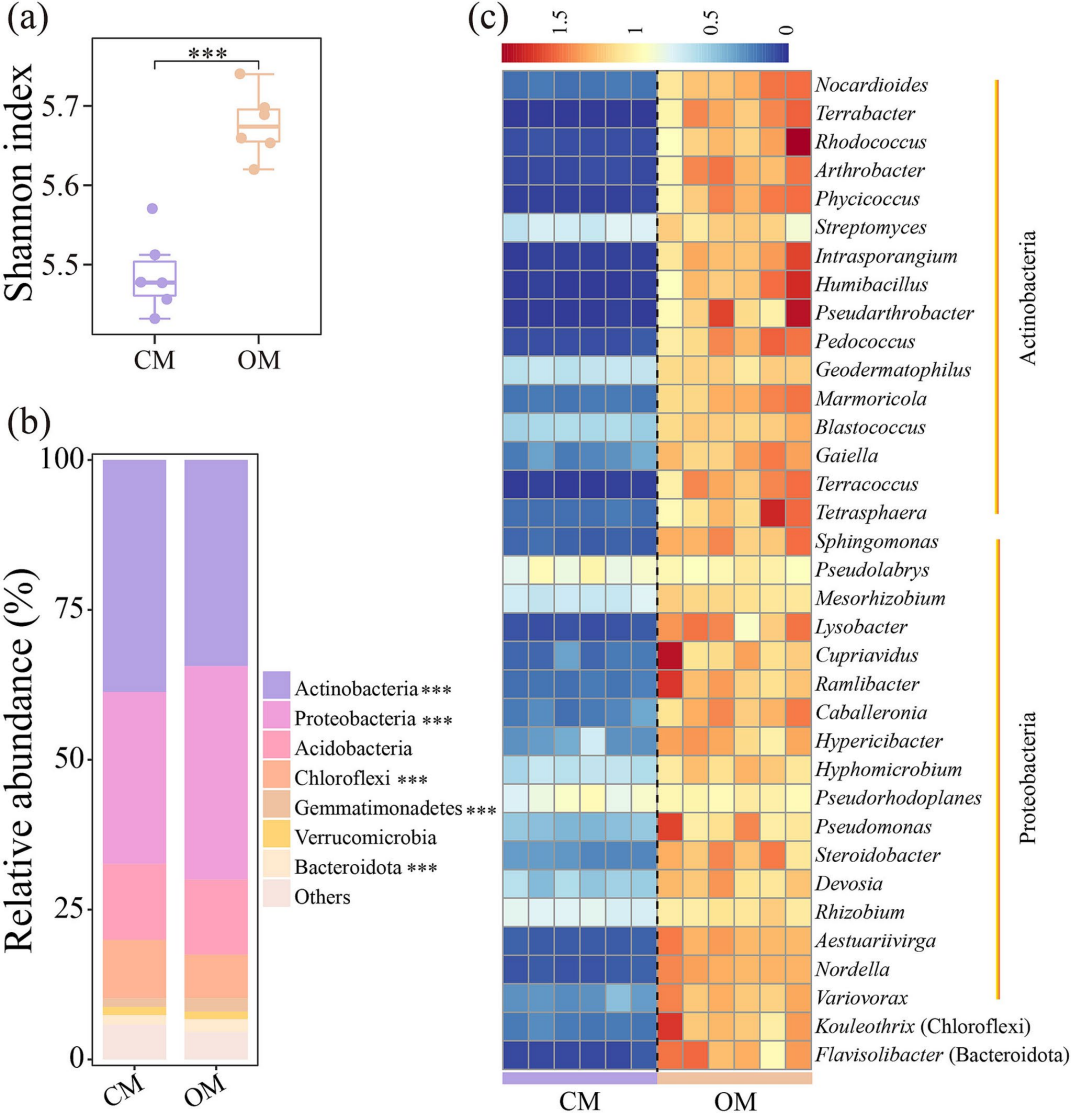
Shannon index **(a)**, relative abundance of abundant phyla **(b)**, and genera with significant differences in relative abundance **(c)** of soil bacterial community in conventional managed (CM) and organic managed (OM) tea plantation soils. Values are means (*n* = 6). ***p < 0.001.

The Shannon index of soil microbial communities in OM was 3.6% higher than that in CM (Figure 1a, *P* < 0.001). Similarly, OM significantly altered soil microbial community composition compared to CM (Figure 1). Actinobacteria, Proteobacteria, Acidobacteria, and Chloroflexi dominanted in abundance at the phylum level, with relative abundance of 31.9–40.7%, 27.7–36.5%, 10.8–14.9%, and 6.8–10.7%, respectively (Figure 1b). OM increased relative abundance of Proteobacteria, Gemmatimonadetes and Bacteroidota by 24.1, 56.6 and 37.4%, while reduced relative abundance of Actinobacteria and Chloroflexi by 12.6 and 34.2% compared to CM (Figure 1b, *P* < 0.001). Further analysis showed OM significantly increased relative abundance of the genera *Nocardioides*, *Terrabacter*, *Rhodococcus*, *Arthrobacter*, *Streptomyces*, *Sphingomonas*, *Pseudolabrys*, *Lysobacter*, and *Pseudomonas* within the changed phyla compared to CM (Figure 1c; Supplementary Table S5, *p* < 0.001).

### 3.3 Abundance and composition of ARGs and VFs

**Figure 2:**
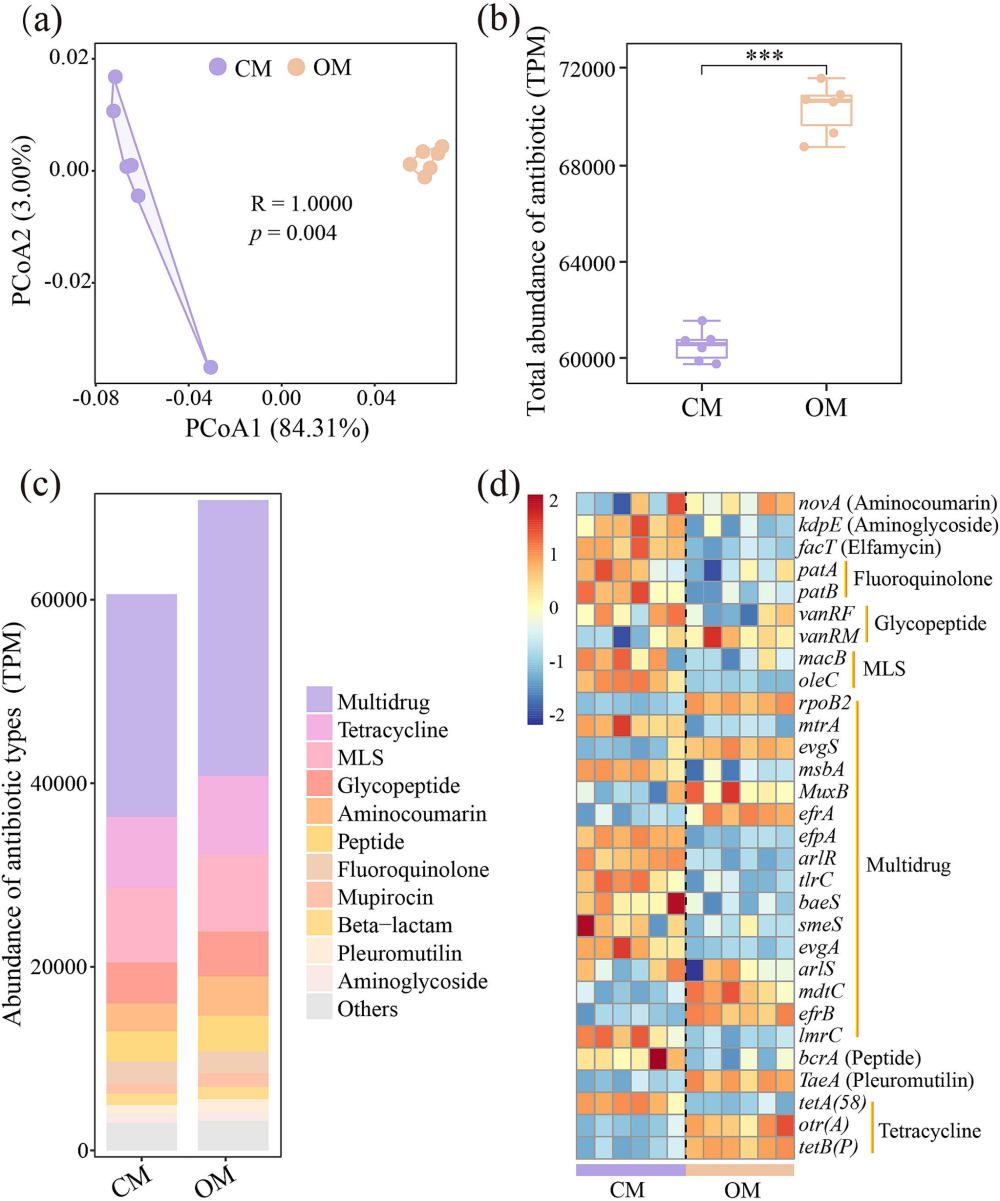
Differences in soil antibiotics resistance genes (ARGs) composition **(a)**, total abundance of ARGs **(b)**, abundance of main antibiotic types identified **(c)**, and differences in abundance of top 30 abundant ARGs subtypes **(d)** in conventional managed (CM) and organic managed (OM) tea plantation soils. Values are means (*n* = 6). ***p < 0.001. The data were standardized (z-score).

PCoA demonstrated that composition of ARGs were significantly separated in OM and CM (Figure 2a, *P* = 0.004). The total abundance of ARGs in OM was 16.9% higher than that in CM (Figure 2b, *P* < 0.001). A total of 21 ARGs and 945 subtypes were detected in OM and CM. Multidrug, Tetracycline, MLS and Glycopeptide were the major ARGs components in all samples, with abundance of 23788.9–30589.7, 7709.7–8851.8, 7681.3–8466.5 and 4324.6–5001.8, respectively (Figure 2c). Among them, OM significantly enriched the abundance of 13 antibiotics (i.e., Multidrug, Tetracycline, MLS, Glycopeptide, Aminocoumarin, Peptide, Mupirocin, Beta-lactam, Pleuromutilin, Fosfomycin, Triclosan, Diaminopyrimidine, and Bicyclomycin), while decreased the abundance of 5 antibiotics (Fluoroquinolone, Aminoglycoside, Rifamycin, Elfamycin, and Nucleoside), compared to CM (Supplementary Table S6). Subsequently, differential analysis on the top abundant 30 subtypes showed that OM enriched relative abundance of 10 subtypes (*rpoB2*, *evgS*, *TaeA*, *MuxB*, *efrA*, *otr(A)*, *tetB(P)*, *mdtC*, *efrB*, and *vanRM*), while decreased relative abundance of 16 subtypes (*macB*, *tetA(58)*, *oleC*, *bcrA*, *mtrA*, *msbA*, *efpA*, *arlR*, *kdpE*, *tlrC*, *baeS*, *facT*, *patA*, *evgA*, *patB*, and *lmrC*), compared to CM (Figure 2d; Supplementary Table S7). Furthermore, the potential host test of ARGs showed that *Mycobacterium* (Unclassified), *Bradyrhizobium* (B. sp.*_35–63-5*), *Streptomyces* (S. sp.*_CEV_2–1*, S. sp.*_ADI95-17*, *S. chartreusis*, S. sp.*_LAM7114* and *S. rishiriensis*), *Saccharopolyspora* (*S. shandongensis* and *S. hirsuta*) and *Actinomadura* (*A. amylolytica* and *A. hibisca*) were shared hosts for both CM and OM. A total of 23 unique hosts were detected in CM, such as *Bacillus* (*B. cereus*), *Dictyobacter* (D. *kobayashii* and *D. aurantiacus*), *Bradyrhizobium* (B. sp.*_35–63-5*) and *Amycolatopsis* (A. *vastitatis* and *A. kentuckyensis*), while 10 unique hosts were detected in OM, such as *Terrabacter* (Terrabacter. sp.*_3264*), *Pseudomonas* (*P. aeruginosa*), *Pseudonocardia* (P. *hierapolitana*), *Tetrasphaera* (T. sp.*_HKS02*) and *Microbispora* (M. sp.*_GKU_823*) (Supplementary Table S8).

**Figure 3:**
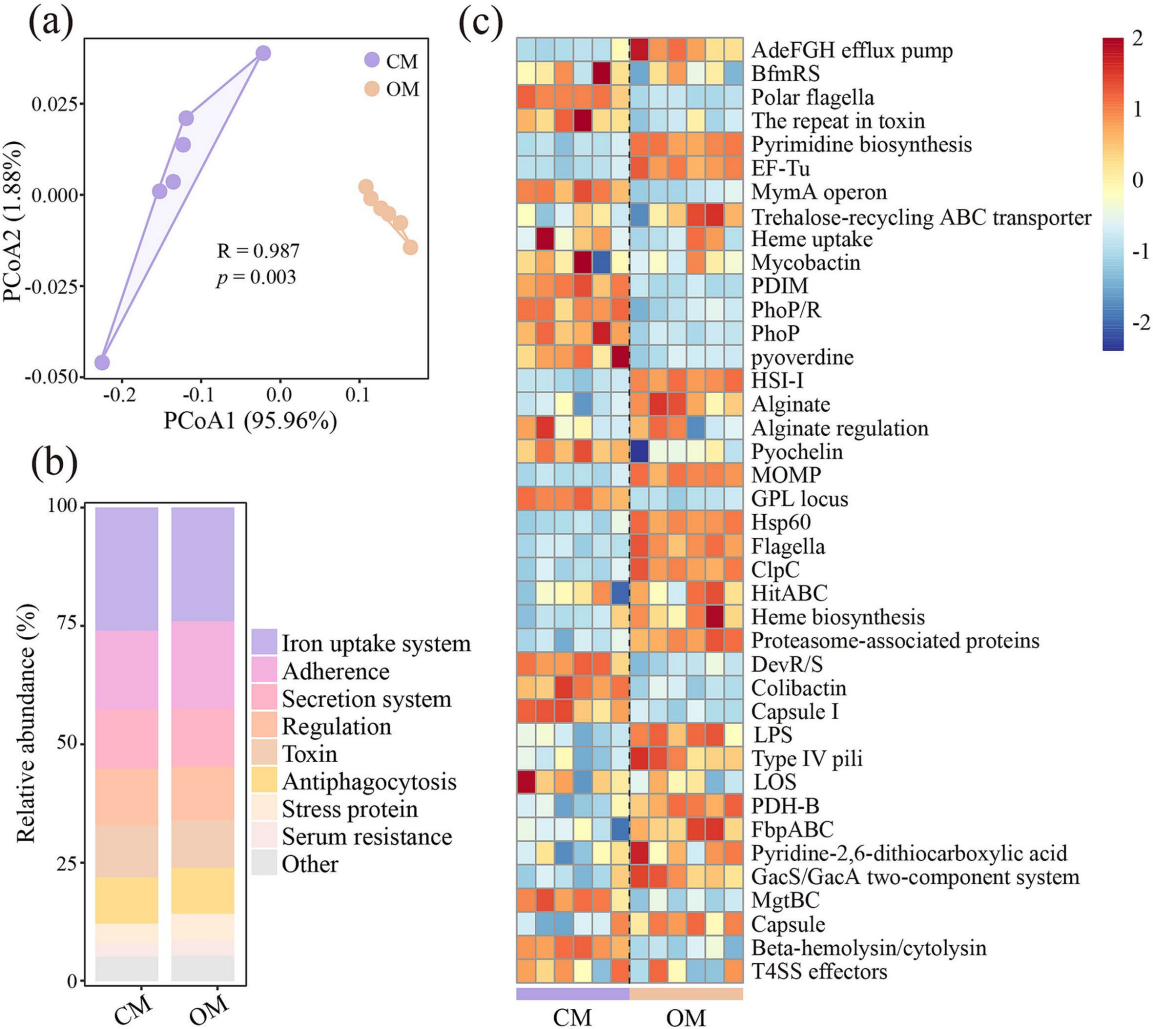
Differences in soil virulence factors (VFs) composition **(a)**, relative abundance of main VFs functions identified **(b)**, and difference in relative abundance of VFs **(c)** in conventional managed (CM) and organic managed (OM) tea plantation soils. Values are means (*n* = 6). The data were standardized (z-score).

PCoA demonstrated that composition of VFs were significantly separated between OM and CM (Figure 3a, *P* = 0.003). Iron uptake system, adherence, secretion systems, regulation, toxin and antiphagocytosis were the dominant encoded functions for both OM and CM, with relative abundance of 23.61–26.55, 16.44–18.67, 12.16–12.71, 11.32–12.26, 9.82–11.21, and 9.53–9.94, respectively (Figure 3b). Among them, OM enriched the relative abundance of adherence, stress protein, serum resistance, phase variation, complement protease, exoenzyme and actin-based motility, while decreased the relative abundance of iron uptake system, secretion system, regulation, toxin and magnesium uptake system, compared to CM (Supplementary Table S9). Further analysis of the top abundant 40 VFs showed that OM significantly increased VFs related to the putative hosts of *Acinetobacter baumannii* (AdeFGH), *Francisella tularensis sub*sp. (repeat in toxin and EF-Tu), *Pseudomonas aeruginosa* (HSI-I and alginate), *Legionella pneumophila sub*sp. (Hsp60), *Pseudomonas stutzeri* (pyridine-2,6-dithiocarboxylic acid), *Pseudomonas syringae pv* (GacS/GacA), *Mycobacterium smegmatis str* (proteasome-associated proteins) (Figure 3c; Supplementary Table S10). However, OM significantly decreased VFs associated with *Aeromonas hydrophila subsp*. (Polar flagella and repeat in toxin), *Mycobacterium sp*. (MymA operon), *Mycobacterium tuberculosis* (PDIM, PhoP/R and PhoP), *Mycobacterium ulcerans* (GPL locus) (Figure 3c; Supplementary Table S10).

### 3.4 Assembly processes and environmental drivers of microbial taxa, ARGs and VFs

**Figure 4:**
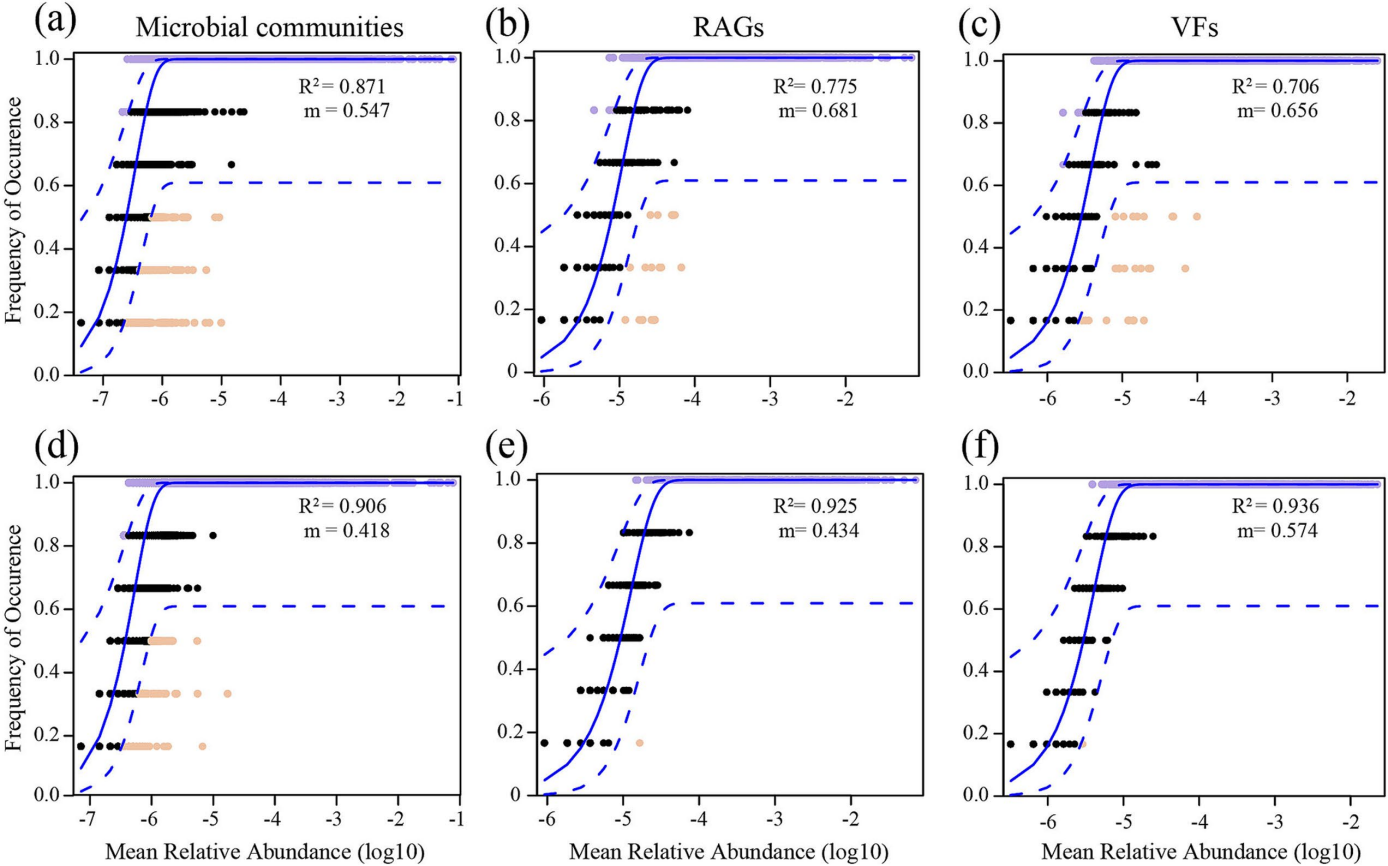
The neutral community model (NCM) of soil microbial communities **(a,d)**, antibiotics resistance genes (ARGs; **b,e**) and virulence factors (VFs; **c,f**) in conventional managed (CM) and organic managed (OM) tea plantation soils. The *R*^2^ value signifies the fitness to the model, and m denotes the migration rate. Solid blue lines indicate optimal fits to the models, with dashed lines representing 95% confidence intervals surrounding the model prediction. Genes deviating from predictions, either occurring more or less frequently, are highlighted in distinct colors.

The neutral community model fitting results showed that the explained variance of soil microbial communities (*R*^2^_OM_ = 0.906, *R*^2^_CM_ = 0.871), ARGs (*R*^2^_OM_ = 0.925, *R*^2^_CM_ = 0.775) and VFs (*R*^2^_OM_ = 0.936, *R*^2^_CM_ = 0.706) in OM was higher than that in CM (Figure 4). These results underscore the important role played of stochastic processes in shaping assembly of soil microbial communities, ARGs and VFs, particularly in OM. We found that OM decreased the m value (the migration rate of community) of soil microbial communities, ARGs and VFs, indicating that the species and gene dispersal was lower, compared to CM (Figure 4).

**Figure 5:**
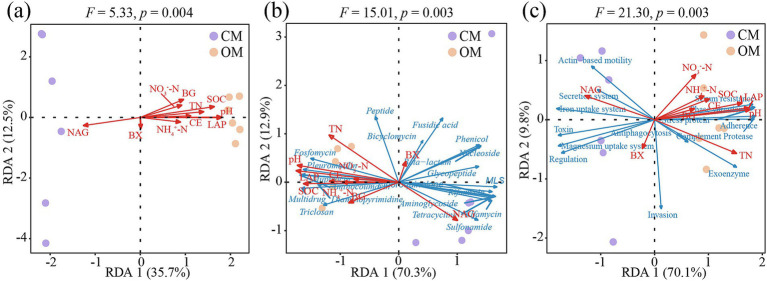
The redundancy analysis (RDA) shows the correlations between soil microbial communities **(a)**, antibiotics resistance genes (ARGs; **b**) and virulence factors (VFs; **c**) with soil properties and enzyme activities in conventional managed (CM) and organic managed (OM) tea plantation soils. Soil properties and enzyme activities are marked with red arrows. Antibiotic types and VFs functions are marked with blue arrows. pH, soil pH; NO_3_^−^-N, nitrate nitrogen; NH_4_^−^-N, ammonium nitrogen; TN, total nitrogen; SOC, soil organic carbon; BG, β-1, 4-glucosidase; NAG, β-1,4-N-acetylglucosaminidase; BX, β-xylosidase; CE, β-cellobiohydrolase; and LAP, L-leucine aminopeptidase.

Soil properties and enzyme activities correlated significantly with composition of soil microbial communities (*F* = 5.33, *p* = 0.004), ARGs (*F* = 15.01, *p* = 0.003) and VFs (*F* = 21.30, *p* = 0.003). RDA result illustrated that RDA1 and RDA2 explained 48.2% of the microbial variations (Figure 5a), 83.2% of the ARGs variations (Figure 5b) and 79.9% of the VFs variations (Figure 5c). Furthermore, soil pH, TN, SOC, NAG and LAP were the main environmental factors driving the composition of soil microbial communities, ARGs and VFs (Supplementary Table S11).

### 3.5 Relationships between microbial taxa, ARGs and VFs

**Figure 6:**
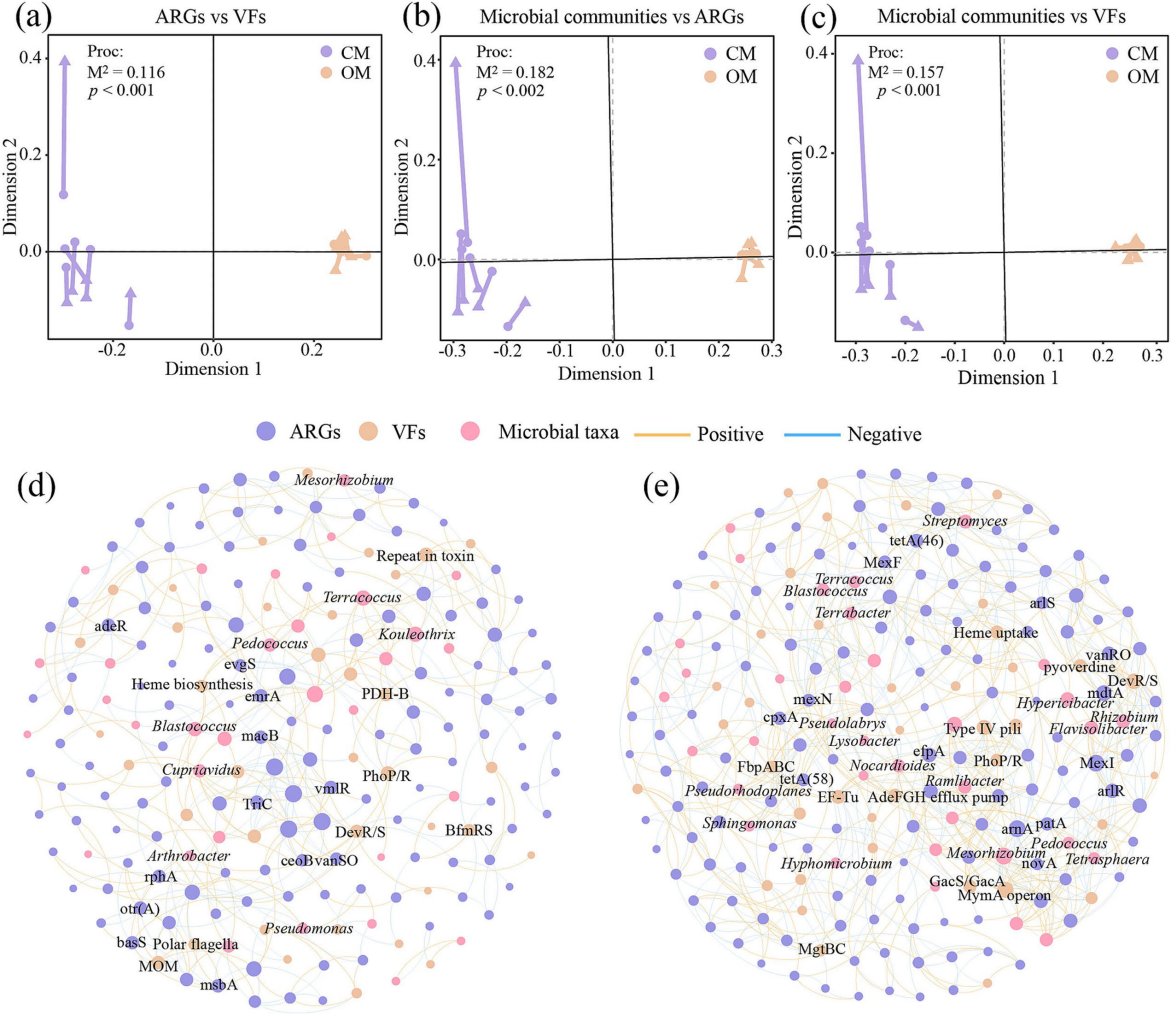
Procrustes analysis based on Bray-Curtis distance reveals the correlations between soil microbial communities **(a)**, antibiotics resistance genes (ARGs; **b**) and virulence factors (VFs; **c**), and co-occurrence network analysis shows the correlations between microbial taxa, ARGs and VFs in conventional managed (CM; **d**) and organic managed (OM; **e**) tea plantation soils based on Pearson coefficient. Nodes with different colors represent different microbial taxa, ARGs and VFs. Orange and blue edges represent positive and negative correlations, respectively.

The Procrustes analysis showed that ARGs and VFs of soil exhibited goodness-of-fit based on the Bray–Curtis dissimilarity metrics (*M*^2^ = 0.116, *p* < 0.001, permutations = 999), indicating significant correlations between ARGs and VFs (Figure 6a). Similarly, Procrustes analysis showed that ARGs (*M*^2^ = 0.182, *p* < 0.002, permutations = 999; Figure 6b) and VFs (*M*^2^ = 0.157, *p* < 0.001, permutations = 999; Figure 6c) correlated significantly with microbial communities, respectively. Networks showed that OM increased the number of node, edge, correlation, average degree, graph density, modularity and average clustering coefficient of the network, while reduced the average path length, compared to CM (Figures 6d,e; Supplementary Table S12). These results suggested that organic management leads to tighter relationships between microbial taxa, ARGs and VFs. Furthermore, OM increased the positive interaction of microbial taxa-ARGs (116 vs. 35), microbial taxa-VFs (40 vs. 21), and ARGs-VFs (119 vs. 57) compared to CM (Supplementary Table S13). Within the network of CM, *Mesorhizobium* (6 subtypes), *Kouleothrix* (5 subtypes) and *Blastococcus* (4 subtypes) were highly correlated with ARGs, and *Cupriavidus* (3 VFs), *Blastococcus* (2 VFs) and *Kouleothrix* (2 VFs) were highly correlated with VFs. Within the network of OM, *Streptomyces* (11 subtypes), *Flavisolibacter* (9 subtypes), *Rhizobium* (8 subtypes) *Blastococcus* (8 subtypes), *Terrabacter* (8 subtypes) and *Nocardioides* (7 subtypes) were highly correlated with ARGs, and *Pseudomonas* (5 VFs), *Hypericibacter* (4 VFs), *Ramlibacter* (4 VFs) and *Hyphomicrobium* (3 VFs) were highly correlated with VFs (Figures 6d,e).

## 4 Discussion

The composition of microbial communities and antibiotic resistance in agricultural soils was closely related to soil health, food production safety and human welfare (Tshikantwa et al., 2018; Bertola et al., 2021). Organic management was reported to increase soil microbial community diversity and change its community structure (Schmidt et al., 2019; Li J. et al., 2022; Shu et al., 2022). This may be attributed to organic matter addition increased organic carbon and available nitrogen contents in the soils (Wu T. et al., 2008), which provided a favorable nutritional environment for microorganisms. On the other hand, organic fertilizer can effectively regulate soil acidification and improve the living environment for soil microorganisms (Chepkorir et al., 2018; Ye et al., 2022), especially in tea plantations. Although organic fertilizer input brings many benefits to the soils, the application of livestock derived organic fertilizers also has the risk of contamination with antibiotics, virulence factors and pathogenic bacteria (Bloem et al., 2017).

In this study, OM significantly changed the ARGs composition and increased its abundance, which was consistent with previous findings (Chen et al., 2016; Sun et al., 2019). Livestock manure contains high levels of antibiotics, ARBs and ARGs (Fang et al., 2014; Wang X. R. et al., 2024), which can potentially spread into the environment when applied to agricultural fields (Joy et al., 2013). We found OM enriched the abundant of Multidrug, Tetracycline, MLS and Glycopeptide antibiotic types compared to CM. Previous studies have confirmed that application of manure introduced extra antibiotics into the agricultural ecosystems (Wang L. et al., 2020; Zhu et al., 2022; Xiao et al., 2023). Further, we explore the differences of main subtypes of the microbial risk genes and OM significantly enriched resistance genes affiliated to multidrug (*rpoB2*, *evgS*, *MuxB* and *efrA*) and Pleuromutilin (*TaeA*). These genes may increase soil resistance to multidrug and Pleuromutilin by encoding efflux pump or antibiotic target alteration (Alcock et al., 2020). In contrast, resistance genes significantly enriched in CM included MLS (*macB* and *oleC*), Tetracycline (*tetA(58)*), Aminocoumarin (*novA*) and Peptide (*bcrA*), and these genes were closely related to antibiotic efflux (Alcock et al., 2020). Virulence factors related to adherence, stress protein, serum resistance, phase variation, complement protease, exoenzyme and actin-based motility were significantly enriched in OM. Bacterial pathogens are able to adhere to host cells by produce a protein or polysaccharide surface layer, and specific enzymes participate in the invasion of host cells and tissues after adhesion (Huang et al., 2016). For intracellular survival, stress proteins affected their persistence and survival (Hingley-Wilson et al., 2010). The significant enrichment of these virulence factors in organically managed agricultural systems suggests that bacterial pathogens may have an enhanced ability to colonize and persist in these environments. Taken together, these results indicated that organic management increased some of ARGs and VFs, which may pose a serious threat to public health (Carmeli et al., 2016; Coll et al., 2018).

Microbial risk depends not only on diversity and abundance of microbial communities, ARGs and VFs, but also on their patterns of coexistence in the same niche (Martínez et al., 2015; Liang et al., 2020). Procrustes analysis and network analysis found significant correlations between microbial communities, ARGs and VFs (Che et al., 2019; Li et al., 2023), and OM increased network complexity (Xie et al., 2018), which may promote the coexistence of microbial taxa, ARGs and VFs thereby increase the risk of microbial contamination (Liang et al., 2020; Zhu et al., 2022). We found that OM increased more positive interactions of microbial taxa-ARGs, microbial taxa-VFs and ARGs-VFs which further supported this conclusion. Within the network, species in CM (such as *Kouleothrix* and *Blastococcus*) and OM (such as *Streptomyces*, *Blastococcus*, *Flavisolibacter*, *Terrabacter*, *Pseudomonas*) were significantly associated with many ARGs and VFs, suggesting that microbial taxa may carry various ARG subtypes and VFs (Yin et al., 2022). It is worth noting that *Streptomyces* (*S. rishiriensis*), *Pseudomonas* (*P. aeruginosa*) and *Terrabacter* (T. sp.*_3264*) with significantly higher relative abundance in OM were identified as potential hosts carrying ARGs, which was consistent with previous findings (Ye et al., 2018; Sheam et al., 2020; Song et al., 2022). Meanwhile, several species affiliated to *Pseudomonas* were potential hosts of VFs in OM, including *P. aeruginosa*, *P. stutzeri* and *P. syringae pv*. The *P. aeruginosa* was a significant pathogen which increased risks of mortality and zoonotic diffusion among patients with sepsis (Dulger, 2020). These results indicate that close links existed between microbial taxa, ARGs and VFs in organic managed tea plantation soils, indicating the significance roles of microbial community succession in growth and spread of ARGs and VFs in soils.

Deterministic and stochastic processes play important roles in assembly of soil microbial communities, ARGs and VFs (Evans et al., 2017; Wang L. et al., 2024; Wang M. M. et al., 2024). Our results support the prominent role of stochastic processes in shaping the assembly of soil microbial communities, ARGs and VFs, particularly in organic managed systems. The higher stochastic assembly from ARGs and VFs in organic managed soils resulted in a more stable antibiotic resistome and virulence factor than that from conventional managed soils (Hou et al., 2021). For soil microbial communities, long-term organic management increased resource availability to reduce resource competitiveness, which resulted in the dominance of stochasticity in soil microbial community assembly process (Badri et al., 2013; Chaparro et al., 2013). To some extent, the regulation principle of ARGs and VFs assembly by environmental stress is similar to regulation of soil microbial community assembly by resources (Liu B. et al., 2022; Liu W. B. et al., 2022). The importance of soil properties on soil microbial communities, ARGs and VFs varies under different management strategies (Cycon et al., 2019; Tang et al., 2023; Wu et al., 2023a). In this study, soil microbial communities, ARGs and VFs were strongly influenced by soil pH, TN, SOC, NAG, and LAP, as proved by previous findings (Zhu et al., 2022; Wu et al., 2023c; Shen et al., 2024). It has been reported that soil pH strongly affected the adsorption and desorption behavior of ARGs (Liu et al., 2010) and organic carbon, total nitrogen, and available potassium altered the distribution of ARGs in soils (Zhu et al., 2022). Furthermore, considering that ARGs and VFs are existed in microbial potential hosts, and the strong correlation between soil properties, enzyme activities and the resistance group may be mediated by soil microbial communities (Li T. T. et al., 2022).

## 5 Conclusion

Our study found that ARGs and VFs could be transferred into soils by agricultural managements in tea plantation. Organic management significantly increased diversity and abundance of ARGs and VFs, and increased relative abundance of microbial hosts harboring ARGs and VFs have significant impacts on soil and human health compared to conventional management. The assembly of microbial communities, ARGs and VFs in organic managed soils was more driven by stochastic processes than that in conventional managed soils. Furthermore, organic management increased the coexistence of microbial taxa-ARGs, microbial taxa-VFs and ARGs-VFs. Taken together, these findings provide more comprehensive insights into the spread, ecological processes and coexistence patterns of ARGs and VFs in tea plantation soils under long-term organic management.

## Data Availability

The datasets presented in this study can be found in online repositories. The names of the repository/repositories and accession number(s) can be found at: https://www.ncbi.nlm.nih.gov/, PRJNA1139650.

## Footnotes

^1^ https://www.omicstudio.cn/tool
