# Supplementary material for: Characterization of antibiotic resistance genes and virulence factors in organic managed tea plantation soils in southwestern China by metagenomics
Source: Front Microbiol. 2025 May 1;16:1580450. doi: 10.3389/fmicb.2025.1580450 (PMC12078288; doi:10.3389/fmicb.2025.1580450)
Supplement: Supplementary file 1 [file Data_Sheet_1.doc]

Table S1 Basic properties and enzyme activity of conventional managed (CM) and organic managed (OM) tea plantation soils.

| Treatment | CM | OM | *p-*value |
| --- | --- | --- | --- |
| pH (H_2_O) | 4.55±0.02 | 5.91±0.06 | 0.001 |
| NO_3_^-^-N (mg/kg) | 17.42±1.55 | 18.93±1.4 | 0.108 |
| NH_4_^+^-N (mg/kg) | 16.09±0.69 | 17.17±1.28 | 0.097 |
| TN (g/kg) | 2.15±0.1 | 2.35±0.07 | 0.002 |
| SOC (g/kg) | 29.85±0.56 | 31.82±0.38 | 0.001 |
| BG (nmol g^-1^ h^-1^) | 96.55±25.21 | 117.38±10.35 | 0.091 |
| BX (nmol g^-1^ h^-1^) | 108.5±14.36 | 88.49±6.5 | 0.011 |
| CE (nmol g^-1^ h^-1^) | 21.81±2.89 | 21.64±4.43 | 0.940 |
| NAG (nmol g^-1^ h^-1^) | 14.97±2.53 | 18.98±3.04 | 0.032 |
| LAP (nmol g^-1^ h^-1^) | 172.35±16.5 | 489.53±14.66 | 0.001 |

Values are means ± standard errors (*n* = 6). pH, soil pH; NO_3_^-^-N, nitrate nitrogen; NH_4_^-^-N, ammonium nitrogen; TN, total nitrogen; SOC, soil organic carbon; BG, β-1, 4-glucosidase; BX, β-xylosidase; CE, β-cellobiohydrolase; NAG, β-1,4-N-acetylglucosaminidase; and LAP, L-leucine aminopeptidase.

Table S2 Overview of sequences merge-ability and number of annotations made for shotgun-metagenome datasets of each sample in conventional managed (CM) and organic managed (OM) tea plantation soils.

| Sample | Raw reads | Raw bases (Gp) | Clean reads | Clean bases (Gp) | Contigs | Contigs bases (Bp) | N50 length (Bp) | N90 length (Bp) |
| --- | --- | --- | --- | --- | --- | --- | --- | --- |
| CM1 | 52,009,734 | 7.85 | 50,682,678 | 7.62 | 550,838 | 322,360,209 | 592 | 345 |
| CM2 | 44,351,672 | 6.70 | 43,258,174 | 6.51 | 408,254 | 241,727,760 | 593 | 344 |
| CM3 | 43,331,356 | 6.54 | 42,232,274 | 6.35 | 390,117 | 232,093,476 | 595 | 344 |
| CM4 | 43,982,636 | 6.64 | 42,819,574 | 6.44 | 394,923 | 229,158,859 | 583 | 343 |
| CM5 | 43,550,494 | 6.58 | 42,411,970 | 6.38 | 353,777 | 210,355,707 | 598 | 345 |
| CM6 | 42,992,792 | 6.49 | 41,963,962 | 6.31 | 346,948 | 193,341,683 | 555 | 340 |
| OM1 | 42,338,954 | 6.39 | 41,318,450 | 6.31 | 233,226 | 110,124,259 | 470 | 329 |
| OM2 | 43,348,742 | 6.65 | 42,129,150 | 6.34 | 260,724 | 126,766,199 | 490 | 332 |
| OM3 | 43,674,334 | 6.59 | 42,222,664 | 6.35 | 246,472 | 120,396,281 | 492 | 332 |
| OM4 | 44,083,000 | 6.66 | 42,686,846 | 6.42 | 265,124 | 132,248,889 | 502 | 334 |
| OM5 | 43,873,536 | 6.62 | 42,597,982 | 6.40 | 277,273 | 137,781,938 | 501 | 334 |
| OM6 | 41,564,510 | 6.28 | 40,534,430 | 6.10 | 239,631 | 114,861,136 | 481 | 332 |

Table S3 Overview of genes catalog assembly to open reading frames (ORFs) of each sample in conventional managed (CM) and organic managed (OM) tea plantation soils.

| Sample | Total length (Bp) | Average length (Bp) | Open reading frames (ORFs) |
| --- | --- | --- | --- |
| CM1 | 282,600,270 | 416.36 | 678,746 |
| CM2 | 211,142,412 | 417.47 | 505,767 |
| CM3 | 202,328,091 | 418.52 | 483,440 |
| CM4 | 199,978,536 | 413.75 | 483,332 |
| CM5 | 183,017,316 | 417.86 | 437,992 |
| CM6 | 168,032,817 | 404.22 | 415,696 |
| OM1 | 98,824,341 | 373.07 | 264,897 |
| OM2 | 113,734,134 | 379.46 | 299,727 |
| OM3 | 107,929,128 | 380.7 | 283,499 |
| OM4 | 118,644,249 | 384.64 | 308,457 |
| OM5 | 123,727,140 | 384.64 | 321,667 |
| OM6 | 103,320,618 | 376.34 | 274,544 |

Table S4 Relative abundance (%) of identified kingdom in conventional managed (CM) and organic managed (OM) tea plantation soils.

| Kingdom | CM | OM | Mean |
| --- | --- | --- | --- |
| Bacteria | 64.28 | 66.30 | 65.29 |
| Archaea | 20.10 | 18.91 | 19.51 |
| Eukaryota | 9.20 | 8.82 | 9.01 |
| Viruses | 2.52 | 3.70 | 3.11 |
| Unknown | 0.40 | 0.40 | 0.40 |

Values are means (*n* = 6).

Table S5 Relative abundance (%) of top 35 microbial genera significantly increased in organic managed (OM) compared with conventional managed (CM) tea plantation soils.

| Genus | Phylum | CM | OM | *p-*value |
| --- | --- | --- | --- | --- |
| *Nocardioides* | Actinobacteria | 0.29±0.016 | 1.91±0.232 | 0.001 |
| *Terrabacter* | Actinobacteria | 0.03±0.006 | 1.68±0.243 | 0.001 |
| *Rhodococcus* | Actinobacteria | 0.09±0.007 | 1.44±0.349 | 0.001 |
| *Arthrobacter* | Actinobacteria | 0.07±0.006 | 1.35±0.195 | 0.001 |
| *Streptomyces* | Actinobacteria | 0.75±0.052 | 1.19±0.138 | 0.001 |
| *Phycicoccus* | Actinobacteria | 0.03±0.005 | 0.84±0.124 | 0.001 |
| *Intrasporangium* | Actinobacteria | 0.02±0.003 | 0.56±0.079 | 0.001 |
| *Humibacillus* | Actinobacteria | 0.01±0.002 | 0.42±0.085 | 0.001 |
| *Pseudarthrobacter* | Actinobacteria | 0.01±0.001 | 0.26±0.067 | 0.001 |
| *Pedococcus* | Actinobacteria | 0.02±0.003 | 0.24±0.037 | 0.001 |
| *Geodermatophilus* | Actinobacteria | 0.11±0.005 | 0.2±0.009 | 0.001 |
| *Marmoricola* | Actinobacteria | 0.03±0.002 | 0.2±0.021 | 0.001 |
| *Blastococcus* | Actinobacteria | 0.08±0.005 | 0.18±0.008 | 0.001 |
| *Gaiella* | Actinobacteria | 0.04±0.009 | 0.17±0.016 | 0.001 |
| *Terracoccus* | Actinobacteria | 0±0.001 | 0.14±0.018 | 0.001 |
| *Tetrasphaera* | Actinobacteria | 0.02±0.002 | 0.13±0.029 | 0.001 |
| *Sphingomonas* | Proteobacteria | 0.38±0.059 | 3.57±0.327 | 0.001 |
| *Pseudolabrys* | Proteobacteria | 1.42±0.142 | 1.59±0.093 | 0.028 |
| *Mesorhizobium* | Proteobacteria | 0.27±0.017 | 0.44±0.017 | 0.001 |
| *Lysobacter* | Proteobacteria | 0.02±0.003 | 0.32±0.053 | 0.001 |
| *Cupriavidus* | Proteobacteria | 0.04±0.014 | 0.26±0.054 | 0.001 |
| *Ramlibacter* | Proteobacteria | 0.03±0.004 | 0.23±0.035 | 0.001 |
| *Caballeronia* | Proteobacteria | 0.04±0.01 | 0.22±0.024 | 0.001 |
| *Hypericibacter* | Proteobacteria | 0.06±0.027 | 0.21±0.023 | 0.001 |
| *Hyphomicrobium* | Proteobacteria | 0.11±0.007 | 0.2±0.016 | 0.001 |
| *Pseudorhodoplanes* | Proteobacteria | 0.16±0.015 | 0.18±0.007 | 0.005 |
| *Pseudomonas* | Proteobacteria | 0.06±0.004 | 0.16±0.032 | 0.001 |
| *Steroidobacter* | Proteobacteria | 0.03±0.004 | 0.15±0.017 | 0.001 |
| *Devosia* | Proteobacteria | 0.06±0.008 | 0.15±0.013 | 0.001 |
| *Rhizobium* | Proteobacteria | 0.1±0.003 | 0.14±0.008 | 0.001 |
| *Aestuariivirga* | Proteobacteria | 0.01±0.001 | 0.13±0.008 | 0.001 |
| *Nordella* | Proteobacteria | 0.01±0.001 | 0.13±0.005 | 0.001 |
| *Variovorax* | Proteobacteria | 0.03±0.006 | 0.12±0.009 | 0.001 |
| *Kouleothrix* | Chloroflexi | 0.04±0.005 | 0.25±0.039 | 0.001 |
| *Flavisolibacter* | Bacteroidota | 0.01±0.002 | 0.11±0.016 | 0.001 |

Values are means ± standard errors (*n* = 6).

Table S6 Abundance of antibiotic class in conventional managed (CM) and organic managed (OM) tea plantation soils.

| Antibiotic class | CM | OM | *p*-value |
| --- | --- | --- | --- |
| Multidrug | 24223.41±600.6 | 30026.54±350.73 | 0.001 |
| Tetracycline | 7829.13±123.96 | 8619.22±139.24 | 0.001 |
| MLS | 8043.28±185.96 | 8335.59±149.69 | 0.018 |
| Glycopeptide | 4467±161.06 | 4901.2±53.15 | 0.001 |
| Aminocoumarin | 3039.54±151.27 | 4292.94±56.03 | 0.001 |
| Peptide | 3266.71±100.99 | 3877.4±69.5 | 0.001 |
| Fluoroquinolone | 2440.27±56.01 | 2347.22±74.59 | 0.039 |
| Mupirocin | 1064.68±30.71 | 1532.56±36.36 | 0.001 |
| Beta-lactam | 1262.77±102.71 | 1317.44±20.1 | 0.224 |
| Pleuromutilin | 915.39±57.8 | 1429.19±51.99 | 0.001 |
| Aminoglycoside | 1004.08±25.17 | 950.96±39.71 | 0.025 |
| Rifamycin | 873.34±64.91 | 796.82±32.19 | 0.032 |
| Elfamycin | 646.57±32.38 | 554.14±32.38 | 0.002 |
| Fosfomycin | 463.19±16.59 | 699.98±37.72 | 0.001 |
| Phenicol | 332.27±34.47 | 329.4±30.76 | 0.879 |
| Triclosan | 252.95±45.06 | 347.29±20 | 0.003 |
| Sulfonamide | 267.97±13.46 | 273.62±12.13 | 0.453 |
| Diaminopyrimidine | 76.91±4.33 | 111.98±10.34 | 0.001 |
| Bicyclomycin | 71.09±8.01 | 82.8±8.33 | 0.037 |
| Nucleoside | 25.67±9.89 | 12.48±4 | 0.017 |
| Fusidic acid | 2.45±5.22 | 0±0 | 0.271 |

Values are means ± standard errors (*n* = 6).

Table S7 Abundance (TPM) of top 30 antibiotics resistance genes (ARGs) in conventional managed (CM) and organic managed (OM) tea plantation soils.

| Antibiotic subtype | Antibiotic class | CM | OM | *p*-value |
| --- | --- | --- | --- | --- |
| *macB* | MLS | 7.4±0.22 | 7.14±0.14 | 0.036 |
| *tetA(58)* | Tetracycline | 6±0.2 | 5.06±0.15 | 0.001 |
| *oleC* | MLS | 3.8±0.16 | 2.99±0.06 | 0.001 |
| *novA* | Aminocoumarin | 3.17±0.11 | 3.23±0.05 | 0.234 |
| *bcrA* | Peptide | 3.1±0.15 | 2.82±0.11 | 0.004 |
| *rpoB2* | Multidrug | 2.17±0.08 | 3.21±0.09 | 0.001 |
| *mtrA* | Multidrug | 2.41±0.07 | 2.13±0.06 | 0.001 |
| *evgS* | Multidrug | 1.73±0.13 | 2.08±0.04 | 0.001 |
| *TaeA* | Pleuromutilin | 1.51±0.08 | 2.02±0.07 | 0.001 |
| *msbA* | Multidrug | 1.72±0.03 | 1.59±0.05 | 0.001 |
| *MuxB* | Multidrug | 1.52±0.07 | 1.62±0.06 | 0.026 |
| *efrA* | Multidrug | 1.26±0.03 | 1.41±0.04 | 0.001 |
| *efpA* | Multidrug | 1.45±0.04 | 1.04±0.05 | 0.001 |
| *arlR* | Multidrug | 1.32±0.03 | 1.05±0.04 | 0.001 |
| *kdpE* | Aminoglycoside | 1.07±0.04 | 0.95±0.04 | 0.001 |
| *tlrC* | Multidrug | 1.05±0.03 | 0.94±0.03 | 0.001 |
| *baeS* | Multidrug | 1.03±0.04 | 0.94±0.03 | 0.002 |
| *smeS* | Multidrug | 1.01±0.06 | 0.95±0.02 | 0.051 |
| *otr(A)* | Tetracycline | 0.79±0.04 | 1.02±0.06 | 0.001 |
| *facT* | Elfamycin | 1.07±0.05 | 0.78±0.05 | 0.001 |
| *patA* | Fluoroquinolone | 0.94±0.06 | 0.86±0.06 | 0.034 |
| *evgA* | Multidrug | 1.01±0.06 | 0.78±0.02 | 0.001 |
| *arlS* | Multidrug | 0.87±0.05 | 0.87±0.06 | 0.820 |
| *tetB(P)* | Tetracycline | 0.7±0.04 | 0.94±0.02 | 0.001 |
| *vanRF* | Glycopeptide | 0.86±0.03 | 0.83±0.04 | 0.080 |
| *mdtC* | Multidrug | 0.71±0.04 | 0.88±0.06 | 0.001 |
| *patB* | Fluoroquinolone | 0.82±0.03 | 0.73±0.03 | 0.001 |
| *efrB* | Multidrug | 0.64±0.02 | 0.8±0.02 | 0.001 |
| *lmrC* | Multidrug | 0.69±0.04 | 0.57±0.02 | 0.001 |
| *vanRM* | Glycopeptide | 0.58±0.05 | 0.66±0.04 | 0.015 |

Values are means ± standard errors (*n* = 6).

Table S8 List of hosts for antibiotics resistance genes (ARGs) in conventional managed (CM) and organic managed (OM) tea plantation soils.

| Treatment | Phylum | Genus | Species |
| --- | --- | --- | --- |
| CM | Actinobacteria | *Herbihabitans* | Herbihabitans_rhizosphaerae |
|  | Actinobacteria | *Mycobacterium* | Unclassified |
|  | Proteobacteria | *Bradyrhizobium* | Unclassified |
|  | Actinobacteria | *Streptomyces* | Streptomyces_sp._CEV_2-1 |
|  | Acidobacteria | *Granulicella* | Granulicella_pectinivorans |
|  | Actinobacteria | *Mycobacterium* | Unclassified |
|  | Actinobacteria | *Mycobacterium* | Unclassified |
|  | Candidatus_Eremiobacteraeota | *Candidatus_Eremiobacter* | Candidatus_Eremiobacter_sp._RRmetagenome_bin22 |
|  | Actinobacteria | *Frankia* | Frankia_discariae |
|  | Actinobacteria | *Actinoallomurus* | Actinoallomurus_bryophytorum |
|  | Actinobacteria | *Mycobacterium* | Unclassified |
|  | Actinobacteria | *Mycobacterium* | Unclassified |
|  | Proteobacteria | *Rhodanobacter* | Rhodanobacter_sp._SCN_66-43 |
|  | Actinobacteria | *Amycolatopsis* | Amycolatopsis_vastitatis |
|  | Proteobacteria | *Rhodanobacter* | Rhodanobacter_sp._SCN_66-43 |
|  | Actinobacteria | *Mycobacterium* | Unclassified |
|  | Actinobacteria | *Mycobacterium* | Unclassified |
|  | Actinobacteria | *Mycobacterium* | Mycobacterium_sp._AB57 |
|  | Proteobacteria | *Bradyrhizobium* | Bradyrhizobium_sp._35-63-5 |
|  | Chloroflexi | *Ktedonobacter* | Ktedonobacter_racemifer |
|  | Actinobacteria | *Mycobacterium* | Unclassified |
|  | Actinobacteria | *Mycobacterium* | Unclassified |
|  | Chloroflexi | *Dictyobacter* | Dictyobacter_kobayashii |
|  | Chloroflexi | *Nitrolancea* | Nitrolancea_hollandica |
|  | Actinobacteria | *Saccharopolyspora* | Saccharopolyspora_shandongensis |
|  | Chloroflexi | *Dictyobacter* | Dictyobacter_aurantiacus |
|  | Actinobacteria | *Mycobacterium* | Unclassified |
|  | Actinobacteria | *Mycobacterium* | Unclassified |
|  | Actinobacteria | *Actinopolymorpha* | Actinopolymorpha_alba |
|  | Actinobacteria | *Streptomyces* | Streptomyces_sp._ADI95-17 |
|  | Proteobacteria | *Rhodanobacter* | Rhodanobacter_sp._C06 |
|  | Proteobacteria | *Mesorhizobium* | Mesorhizobium_soli |
|  | Actinobacteria | *Mycobacterium* | Unclassified |
|  | Actinobacteria | *Marmoricola* | Marmoricola_solisilvae |
|  | Actinobacteria | *Streptomyces* | Streptomyces_chartreusis |
|  | Actinobacteria | *Mycobacterium* | Unclassified |
|  | Actinobacteria | *Mycobacterium* | Unclassified |
|  | Chloroflexi | *Ktedonobacter* | Ktedonobacter_racemifer |
|  | Firmicutes | *Moorella* | Moorella_thermoacetica |
|  | Proteobacteria | *Roseiarcus* | Roseiarcus_fermentans |
|  | Actinobacteria | *Mycobacterium* | Unclassified |
|  | Chloroflexi | *Nitrolancea* | Nitrolancea_hollandica |
|  | Chloroflexi | *Dictyobacter* | Dictyobacter_aurantiacus |
|  | Actinobacteria | *Asanoa* | Asanoa_hainanensis |
|  | Actinobacteria | *Frankia* | Frankia_discariae |
|  | Actinobacteria | *Mycobacterium* | Unclassified |
|  | Proteobacteria | *Bradyrhizobium* | Unclassified |
|  | Firmicutes | *Bacillus* | Bacillus_aciditolerans |
|  | Proteobacteria | *Roseiarcus* | Roseiarcus_fermentans |
|  | Actinobacteria | *Mycobacterium* | Unclassified |
|  | Actinobacteria | *Mycobacterium* | Unclassified |
|  | Actinobacteria | *Mycobacterium* | Mycobacterium_ahvazicum |
|  | Actinobacteria | *Gaiella* | Gaiella_sp._SCGC_AG-212-M14 |
|  | Actinobacteria | *Amycolatopsis* | Amycolatopsis_vastitatis |
|  | Actinobacteria | *Mycobacterium* | Unclassified |
|  | Chloroflexi | *Dictyobacter* | Dictyobacter_kobayashii |
|  | Actinobacteria | *Mycobacterium* | Unclassified |
|  | Firmicutes | *Bacillus* | Unclassified |
|  | Actinobacteria | *Actinoallomurus* | Actinoallomurus_bryophytorum |
|  | Firmicutes | *Bacillus* | Bacillus_cereus |
|  | Firmicutes | *Bacillus* | Bacillus_cereus |
|  | Actinobacteria | *Herbiconiux* | Herbiconiux_solani |
|  | Firmicutes | *Bacillus* | Unclassified |
|  | Actinobacteria | *Mycobacterium* | Unclassified |
|  | Firmicutes | *Bacillus* | Unclassified |
|  | Firmicutes | *Bacillus* | Bacillus_cereus |
|  | Firmicutes | *Bacillus* | Bacillus_cereus |
|  | Firmicutes | *Bacillus* | Bacillus_cereus |
|  | Firmicutes | *Bacillus* | Unclassified |
|  | Proteobacteria | *Hypericibacter* | Hypericibacter_adhaerens |
|  | Actinobacteria | *Humibacter* | Humibacter_sp._BT305 |
|  | Firmicutes | *Bacillus* | Bacillus_cereus |
|  | Firmicutes | *Bacillus* | Bacillus_cereus |
|  | Firmicutes | *Bacillus* | Bacillus_cereus |
|  | Firmicutes | *Bacillus* | Bacillus_cereus |
|  | Firmicutes | *Bacillus* | Bacillus_cereus |
|  | Firmicutes | *Bacillus* | Bacillus_cereus |
|  | Firmicutes | *Bacillus* | Bacillus_cereus |
|  | Firmicutes | *Bacillus* | Bacillus_cereus |
|  | Firmicutes | *Bacillus* | Bacillus_cereus |
|  | Firmicutes | *Bacillus* | Bacillus_cereus |
|  | Firmicutes | *Bacillus* | Bacillus_cereus |
|  | Firmicutes | *Bacillus* | Bacillus_cereus |
|  | Actinobacteria | *Frankia* | Frankia_discariae |
|  | Proteobacteria | *Desulfosarcina* | Desulfosarcina_widdelii |
|  | Firmicutes | *Bacillus* | Bacillus_cereus |
|  | Firmicutes | *Bacillus* | Bacillus_cereus |
|  | Firmicutes | *Bacillus* | Unclassified |
|  | Firmicutes | *Bacillus* | Bacillus_cereus |
|  | Firmicutes | *Bacillus* | Bacillus_cereus |
|  | Firmicutes | *Bacillus* | Bacillus_cereus |
|  | Firmicutes | *Bacillus* | Unclassified |
|  | Firmicutes | *Bacillus* | Bacillus_cereus |
|  | Firmicutes | *Bacillus* | Bacillus_cereus |
|  | Firmicutes | *Bacillus* | Bacillus_sp._72 |
|  | Firmicutes | *Bacillus* | Bacillus_cereus |
|  | Actinobacteria | *Mycobacterium* | Unclassified |
|  | Firmicutes | *Bacillus* | Bacillus_cereus |
|  | Firmicutes | *Bacillus* | Bacillus_cereus |
|  | Actinobacteria | *Actinomadura* | Actinomadura_amylolytica |
|  | Actinobacteria | *Mycobacterium* | Unclassified |
|  | Actinobacteria | *Streptomyces* | Unclassified |
|  | Actinobacteria | *Streptomyces* | Streptomyces_sp._LAM7114 |
|  | Actinobacteria | *Mycobacterium* | Mycobacterium_sp. |
|  | Actinobacteria | *Streptomyces* | Unclassified |
|  | Actinobacteria | *Amycolatopsis* | Amycolatopsis_vastitatis |
|  | Actinobacteria | *Gaiella* | Gaiella_sp._SCGC_AG-212-M14 |
|  | Actinobacteria | *Mycobacterium* | Unclassified |
|  | Chloroflexi | *Nitrolancea* | Nitrolancea_hollandica |
|  | Actinobacteria | *Amycolatopsis* | Amycolatopsis_kentuckyensis |
|  | Actinobacteria | *Agromyces* | Agromyces_cerinus |
|  | Chloroflexi | *Dictyobacter* | Dictyobacter_aurantiacus |
|  | Proteobacteria | *Bradyrhizobium* | Bradyrhizobium_sp._35-63-5 |
| OM | Actinobacteria | *Mycobacterium* | Unclassified |
|  | Proteobacteria | *Pseudomonas* | Pseudomonas_aeruginosa |
|  | Actinobacteria | *Jiangella* | Jiangella_asiatica |
|  | Actinobacteria | *Actinomadura* | Actinomadura_hibisca |
|  | Actinobacteria | *Microbispora* | Microbispora_sp._GKU_823 |
|  | Actinobacteria | *Saccharopolyspora* | Saccharopolyspora_hirsuta |
|  | Actinobacteria | *Saccharopolyspora* | Saccharopolyspora_shandongensis |
|  | Actinobacteria | *Pseudonocardia* | Pseudonocardia_hierapolitana |
|  | Actinobacteria | *Terrabacter* | Terrabacter_sp._3264 |
|  | Actinobacteria | *Kibdelosporangium* | Kibdelosporangium_sp._MJ126-NF4 |
|  | Proteobacteria | *Dokdonella* | Dokdonella_fugitiva |
|  | Actinobacteria | *Mycobacterium* | Unclassified |
|  | Proteobacteria | *Bradyrhizobium* | Bradyrhizobium_sp._35-63-5 |
|  | Chloroflexi | *Herpetosiphon* | Herpetosiphon_llansteffanensis |
|  | Actinobacteria | *Streptomyces* | Streptomyces_rishiriensis |
|  | Actinobacteria | *Rhodococcus* | Rhodococcus_wratislaviensis |
|  | Actinobacteria | *Mycobacterium* | Mycobacterium_sp._852002-40037_SCH5390672 |
|  | Actinobacteria | *Tetrasphaera* | Tetrasphaera_sp._HKS02 |

Table S9 Relative abundance (%) of virulence factors (VFs) types in conventional managed (CM) and organic managed (OM) tea plantation soils.

| VFs types | CM | OM | *p-*value |
| --- | --- | --- | --- |
| Iron uptake system | 25.98±0.44 | 24±0.36 | 0.001 |
| Adherence | 16.69±0.15 | 18.37±0.23 | 0.001 |
| Secretion system | 12.48±0.22 | 12.29±0.1 | 0.078 |
| Regulation | 12.03±0.2 | 11.38±0.05 | 0.001 |
| Toxin | 10.98±0.2 | 9.99±0.15 | 0.001 |
| Antiphagocytosis | 9.72±0.15 | 9.78±0.14 | 0.498 |
| Stress protein | 4.09±0.12 | 5.51±0.15 | 0.001 |
| Serum resistance | 2.93±0.08 | 3.38±0.08 | 0.001 |
| Invasion | 2.83±0.07 | 2.83±0.06 | 0.918 |
| Magnesium uptake system | 1.3±0.06 | 1.04±0.03 | 0.001 |
| Phase variation | 0.89±0.03 | 1.15±0.02 | 0.001 |
| Complement Protease | 0.03±0.01 | 0.16±0.01 | 0.001 |
| Exoenzyme | 0.05±0.04 | 0.11±0.01 | 0.004 |
| Actin-based motility | 0.001±0.0006 | 0.0003±0.0006 | 0.065 |

Values are means ± standard errors (*n* = 6).

Table S10 Relative abundance (%) of top 40 virulence factors (VFs) in conventional managed (CM) and organic managed (OM) tea plantation soils.

| VFs | Genus | Species | CM | OM | *p-*value |
| --- | --- | --- | --- | --- | --- |
| AdeFGH efflux pump | Acinetobacter | Acinetobacter baumannii | 1.31±0.05 | 1.55±0.08 | 0.001 |
| BfmRS | Acinetobacter | Acinetobacter baumannii | 0.85±0.03 | 0.82±0.03 | 0.197 |
| Polar flagella | Aeromonas | Aeromonas hydrophila subsp. | 1.96±0.07 | 1.52±0.03 | 0.001 |
| Repeat in toxin | Aeromonas | Aeromonas hydrophila subsp. | 0.84±0.02 | 0.79±0.01 | 0.001 |
| Pyrimidine biosynthesis | Francisella | Francisella tularensis subsp. | 0.96±0.03 | 1.26±0.03 | 0.001 |
| EF-Tu | Francisella | Francisella tularensis subsp. | 0.63±0.02 | 0.88±0.03 | 0.001 |
| MymA operon | Mycobacterium | Mycobacterium sp. | 2.11±0.06 | 1.78±0.04 | 0.001 |
| Trehalose-recycling ABC transporter | Mycobacterium | Mycobacterium sp. | 1.33±0.03 | 1.37±0.06 | 0.186 |
| Heme uptake | Mycobacterium | Mycobacterium sp. | 0.7±0.04 | 0.68±0.04 | 0.352 |
| Mycobactin | Mycobacterium | Mycobacterium tuberculosis | 0.9±0.03 | 0.89±0.01 | 0.618 |
| PDIM | Mycobacterium | Mycobacterium tuberculosis | 1.33±0.03 | 1.11±0.02 | 0.001 |
| PhoP/R | Mycobacterium | Mycobacterium tuberculosis | 1.29±0.02 | 1.17±0.02 | 0.001 |
| PhoP | Mycobacterium | Mycobacterium tuberculosis | 0.89±0.03 | 0.79±0.01 | 0.001 |
| pyoverdine | Pseudomonas | Pseudomonas aeruginosa | 2.35±0.25 | 1.74±0.07 | 0.001 |
| HSI-I | Pseudomonas | Pseudomonas aeruginosa | 1.7±0.02 | 1.92±0.02 | 0.001 |
| Alginate | Pseudomonas | Pseudomonas aeruginosa | 1.15±0.05 | 1.3±0.05 | 0.001 |
| Alginate regulation | Pseudomonas | Pseudomonas aeruginosa | 1.12±0.02 | 1.12±0.03 | 0.835 |
| Pyochelin | Pseudomonas | Pseudomonas aeruginosa | 0.7±0.08 | 0.65±0.03 | 0.063 |
| MOMP | Chlamydia | Chlamydia trachomatis | 0.67±0.03 | 1±0.03 | 0.001 |
| GPL locus | Mycobacterium | Mycobacterium ulcerans | 1.77±0.06 | 1.25±0.03 | 0.001 |
| Hsp60 | Legionella | Legionella pneumophila subsp. | 0.81±0.04 | 1.12±0.03 | 0.001 |
| Flagella | Helicobacter | Helicobacter pylori | 0.65±0.01 | 0.77±0.02 | 0.001 |
| ClpC | Listeria | Listeria monocytogenes | 1.35±0.07 | 1.83±0.06 | 0.001 |
| HitABC | Haemophilus | Haemophilus influenzae | 1.19±0.04 | 1.22±0.03 | 0.136 |
| Heme biosynthesis | Haemophilus | Haemophilus somnus | 0.85±0.03 | 0.92±0.03 | 0.003 |
| Proteasome-associated proteins | Mycobacterium | Mycobacterium smegmatis str | 0.58±0.03 | 0.73±0.02 | 0.001 |
| DevR/S | Mycobacterium | Mycobacterium vanbaalenii | 0.81±0.04 | 0.59±0.04 | 0.001 |
| Colibactin | Klebsiella | Klebsiella pneumoniae subsp. | 1.23±0.05 | 1±0.03 | 0.001 |
| Capsule I | Burkholderia | Burkholderia thailandensis E264 | 0.7±0.04 | 0.57±0.01 | 0.001 |
| LPS | Brucella | Brucella melitensis bv | 1.03±0.03 | 1.14±0.04 | 0.001 |
| Type IV pili | Yersinia | Yersinia enterocolitica subsp. | 1.47±0.05 | 1.63±0.05 | 0.001 |
| LOS | Campylobacter | Campylobacter jejuni subs. | 1.36±0.04 | 1.33±0.03 | 0.267 |
| PDH-B | Mycoplasma | Mycoplasma agalactiae | 0.55±0.03 | 0.63±0.02 | 0.001 |
| FbpABC | Neisseria | Neisseria meningitidis | 2.07±0.04 | 2.18±0.04 | 0.001 |
| Pyridine-2,6-dithiocarboxylic acid | Pseudomonas | Pseudomonas stutzeri | 0.63±0.02 | 0.66±0.02 | 0.047 |
| GacS/GacA | Pseudomonas | Pseudomonas syringae pv | 0.61±0.04 | 0.72±0.04 | 0.001 |
| MgtBC | Salmonella | Salmonella enterica subsp. | 0.63±0.02 | 0.55±0.02 | 0.001 |
| Capsule | Staphylococcus | Staphylococcus aureus subsp. | 0.58±0.02 | 0.62±0.01 | 0.010 |
| Beta-hemolysin/cytolysin | Streptococcus | Streptococcus agalactiae | 2.23±0.03 | 1.94±0.06 | 0.001 |
| T4SS effectors | Coxiella | Coxiella burnetii | 0.54±0.02 | 0.53±0.03 | 0.359 |

Values are means ± standard errors (*n* = 6).

Table S11 Effects of soil properties and enzyme activities on soil microbial communities, antibiotics resistance genes (ARGs) and virulence factors (VFs).

| Type |  | RDA1 | RDA2 | r^2^ | *p*-value |
| --- | --- | --- | --- | --- | --- |
| Microbial  community | pH | 0.99996182 | 0.008738336 | 0.9927 | 0.002 |
|  | NO_3_^-^-N | 0.914838373 | 0.403820196 | 0.3102 | 0.197 |
|  | NH4^-^-N | 0.986266834 | -0.165159719 | 0.2504 | 0.244 |
|  | TN | 0.990851168 | 0.134959115 | 0.6181 | 0.014 |
|  | SOC | 0.976786788 | 0.214213841 | 0.8674 | 0.002 |
|  | BG | 0.849886793 | 0.526965311 | 0.3919 | 0.135 |
|  | NAG | -0.977908138 | -0.209035101 | 0.5101 | 0.048 |
|  | BX | 0.027774762 | -0.999614207 | 0.041 | 0.818 |
|  | CE | 0.998831047 | 0.048337755 | 0.3848 | 0.134 |
|  | LAP | 0.999995626 | -0.002957692 | 0.9933 | 0.001 |
| ARGs | pH | -0.980266827 | 0.197678901 | 0.9627 | 0.003 |
|  | NO_3_^-^-N | -0.996287283 | 0.086090943 | 0.1829 | 0.391 |
|  | NH4^-^-N | -0.999987225 | -0.005054597 | 0.2831 | 0.262 |
|  | TN | -0.774933297 | 0.632043025 | 0.7275 | 0.006 |
|  | SOC | -0.999820619 | -0.018940156 | 0.8152 | 0.003 |
|  | BG | -0.895838235 | -0.444380305 | 0.2794 | 0.241 |
|  | NAG | 0.787827225 | -0.615896309 | 0.5023 | 0.042 |
|  | BX | 0.27262768 | 0.962119612 | 0.0611 | 0.807 |
|  | CE | -0.999838605 | 0.01796564 | 0.4306 | 0.083 |
|  | LAP | -0.990971903 | 0.13406971 | 0.9622 | 0.002 |
| VFs | pH | 0.995602641 | 0.093677002 | 0.9747 | 0.001 |
|  | NO_3_^-^-N | 0.710338607 | 0.703860116 | 0.3592 | 0.145 |
|  | NH4^-^-N | 0.918131725 | 0.396275328 | 0.3064 | 0.207 |
|  | TN | 0.938037455 | -0.346533883 | 0.81 | 0.002 |
|  | SOC | 0.985723745 | 0.168370717 | 0.8356 | 0.001 |
|  | BG | 0.970971072 | 0.239196944 | 0.1783 | 0.403 |
|  | NAG | -0.954925611 | 0.296845207 | 0.5619 | 0.032 |
|  | BX | -0.423621436 | -0.905839323 | 0.087 | 0.671 |
|  | CE | 0.946471235 | 0.322788167 | 0.3563 | 0.135 |
|  | LAP | 0.993762601 | 0.111516333 | 0.956 | 0.002 |

pH, soil pH; NO_3_^-^-N, nitrate nitrogen; NH_4_^-^-N, ammonium nitrogen; TN, total nitrogen; SOC, soil organic carbon; BG, β-1, 4-glucosidase; BX, β-xylosidase; CE, β-cellobiohydrolase; NAG, β-1,4-N-acetylglucosaminidase; and LAP, L-leucine aminopeptidase.

Table S12 Topological characteristics of co-occurrence networks in conventional managed (CM) and organic managed (OM) tea plantation soils.

| Network characteristic | CM | OM |
| --- | --- | --- |
| Number of nodes | 200 | 214 |
| Number of edges | 427 | 784 |
| Number of positive correlations | 226 | 399 |
| Number of negative correlations | 201 | 385 |
| Average degree (avgK) | 4.27 | 7.327 |
| Average weighted degree | 0.468 | 0.259 |
| Network diameter | 17 | 10 |
| Graph density | 0.021 | 0.034 |
| Modularity (M) | 7.352 | 7.949 |
| Interconnecting piece | 4 | 1 |
| Average clustering coefficient (avgCC) | 0.106 | 0.388 |
| Average path length (APL) | 7.135 | 4.667 |

Table S13 The number of interactions within and among soil microbial taxa antibiotics resistance genes (ARGs) and virulence factors (VFs) in conventional managed (CM) and organic managed (OM) tea plantation soils.

| Interaction | CM | OM |
| --- | --- | --- |
| Microbial taxa—Microbial taxa | 17 (P: 16/N: 1) | 34 (P: 28/N: 6) |
| Microbial taxa—ARGs | 83 (P: 35/N: 48) | 172 (P: 116/N: 56) |
| Microbial taxa—VFs | 28 (P: 21/N: 7) | 59 (P: 40/N: 19) |
| ARGs—ARGs | 181 (P: 91/N: 90) | 317 (P: 160/N: 157) |
| ARGs—VFs | 107 (P: 57/N: 50) | 179 (P: 119/N: 60) |
| VFs—VFs | 11 (P: 6/N: 5) | 23 (P: 12/N: 11) |

Numbers outside parentheses represent the sum of positive and negative edge numbers. P and N inside parentheses represent positive correlation and negative correlation, respectively.
